# Reducing Choice-Blindness? An Experimental Study Comparing Experienced Meditators to Non-Meditators

**DOI:** 10.3390/ejihpe12110113

**Published:** 2022-11-06

**Authors:** Léa Lachaud, Baptiste Jacquet, Jean Baratgin

**Affiliations:** 1 CHArt RNSR 200515259U, Université Paris 8, 93200 Saint-Denis, France; 2 Lutin Userlab, Université Paris 8, 75930 Paris, France; 3 P-A-R-I-S Association, 75005 Paris, France

**Keywords:** mindfulness, experienced meditators, meditation effects, choice-blindness, decision-making

## Abstract

The mindfulness trait is an intrinsic characteristic of one’s disposition that facilitates awareness of the present moment. Meditation has proven to enhance situational awareness. In this study, we compared the performance of participants that were split into two groups depending on their experience in mindfulness meditation (a control group naive to mindfulness meditation and a group of experienced mindfulness meditators). Choice-blindness happens when people fail to notice mismatches between their intentions and the consequences of decisions. Our task consisted of decisions where participants chose one preferred female facial image from a pair of images for a total of 15 decisions. By reversing the decisions, unbeknownst to the participants, three discrepancies were introduced in an online experimental design. Our results indicate that the likelihood of detecting one or more manipulations was higher in the mindful group compared to the control group. The higher FMI scores of the mindful group did not contribute to this observation; only the practice of mindfulness meditation itself did. Thus, this could be explained by better introspective access and control of reasoning processes acquired during practice and not by the latent characteristics that are attributed to the mindfulness trait.

## 1. Introduction

Cognitive biases are responsible for many of the behaviors that deviate from what would be considered rational in a given situation. Research has shown evidence supporting the idea that mindfulness meditation could reduce the effect of some well-known cognitive biases ([1,2,3,4]; for examples).

Given that studies suggest that regular mindfulness practice improves introspective access [5,6] and the ability to focus attention on the details of the environment [7,8]; it may reduce biases initially caused by the lack of sufficient introspection and poor attention to the environment.

The choice-blindness (CB) effect, first observed by Johansson and collaborators [9], is one such bias. This study reveals the difficulty participants face in detecting manipulations of their initial choices by an experimenter who replaced the picture participants had chosen with one they had not. Some authors have suggested that this effect could be the result of insufficient introspection [10,11,12] or an underestimation of the influence of environmental and situational factors [13,14,15,16,17].

According to Petitmengin and collaborators [18], the CB effect is caused by blindness to the decision processes (non-conscious reasons for our choices) and not by blindness to the decision criteria (conscious explanation of our choices). Indeed, their study showed that the detection rate of manipulations in a CB context was improved following an elicitation interview conducted just after the presentation of the non-chosen object (photograph). Manipulated trials followed by an elicitation interview were detected in 80% of the cases, whereas classic manipulated trials (not followed by the interview) were only detected in 33% of the cases. The explanatory interview method [19,20] aims to provoke in the subject, via specific questions, an awareness of subjective experience that is usually not directly accessible to consciousness (“pre-reflective” awareness) [18]. However, this method has an important point in common with the activity of mindfulness meditation: that of performing introspective acts of recalling and directing attention in order to increase awareness of the process of choice [18]. Furthermore, the authors [18] note that the sole act of evoking subjective experience through an explanatory interview does not improve the detection of manipulations that have not been followed by this interview. The authors then suggest that these introspective abilities are skills that need to be learned, which seems to be achievable through the regular practice of mindfulness [5,6].

The results of this research indicate that studying the effect of CB on experienced mindfulness meditators may be a relevant direction to follow. In doing so, we could investigate whether this effect can be found in participants who have acquired abilities similar to those triggered by the explanatory interviews. For this reason, experienced mindfulness meditators should be able to avoid the CB effect with greater ease than inexperienced meditators. Should this hypothesis be correct, it could have consequences in the field of decision-making.

This article highlights the literature indicating a potential link between a decrease in CB and mindfulness meditation and replicates the study of Johansson and collaborators [9] with a group of experienced meditators and a control group not experienced in meditation to investigate whether being experienced in the practice of mindfulness meditation does indeed have a noticeable effect on CB.

### 1.1. The Practice of Mindfulness Meditation and Its Cognitive Effects

Mindfulness consists of both a natural psychological predisposition as well as a personality trait that is developed and maintained through the practice of mindfulness meditation [21]. The practice of mindfulness consists of focusing one’s attention and awareness on the present moment by adopting a non-judgmental attitude [22,23]. This practice is mainly characterized by two attitudes: (i) the self-regulation of attention to maintain a context of immediacy for a given situation, thereby allowing for the increased recognition of mental events in the present moment and (ii) adopting a particular mental orientation characterized by curiosity and acceptance of one’s experiences in the present moment, while remaining open to alternate interpretations with a goal of objectivity [24].

Tang and collaborators present a model of mindfulness meditation including at least three components: “enhanced attention control, improved emotion regulation, and altered self-awareness (diminished self-referential processing and enhanced body awareness)”. The interaction between these three factors enhances self-regulation [25] (p. 2).

Others describe mindfulness as a specific form of mental training to develop self-awareness, self-regulation, and self-transcendence [26] and as a mental training associated with activating emotional flexibility, cognitive flexibility, and attention processes [27]. Regular use of these aptitudes by experienced meditators allows the “mindfulness” personality trait to develop and allows modulating networks of self-processing to reduce cognitive bias.

### 1.2. The “Mindful” Personality Trait: A Possible Consequence of Mindfulness Practice

Studies have shown that people who regularly meditate often develop trait, or dispositional, mindfulness [28]. The mindfulness trait yields the ability to dedicate attention to the current moment, enabling greater situational awareness as well as an improved ability to take a step back and observe one’s own thoughts [29]. This ability remains stable over time [21] and can be maintained or improved by the practice of a meditative activity [30]. People who have acquired the mindfulness trait thanks to the consistent practice of mindfulness meditation are more likely to possess a habit of observing their thoughts and behaviors with greater openness and objectivity compared to the general population who is naive to meditation [3]. For example, experienced meditators are able to detect light flashes of shorter duration than the non-meditators [31]. These results are interpreted as the consequence of “an enduring increase in sensitivity, perhaps the long-term effect of the practice of mindfulness meditation on certain perceptual habit patterns” [31] (p. 727).

Trait mindfulness can also decrease the influence of some cognitive biases related to decision-making [1,2,3,4,32] by placing meditators in an analytical position to observe their environment and their own bodily and mental events [3]. It can also improve working memory capacity by decreasing mind-wandering [33], keeping one’s attention focused on the present moment [1], improving one’s ability to explore one’s own values and priorities [3] and by improving the ability to regulate emotional states recognized to be influencing decision-making [34,35,36]. Carlson also listed several pieces of empirical evidence supporting the hypothesis that mindfulness would improve self-knowledge of emotions, thoughts, behavior and the accuracy of beliefs regarding metaperception [37].

Trait mindfulness is often measured with a scale called the Freiburg Mindfulness Inventory (FMI). The version *FMI-14 items* is validated for use outside of a Buddhist context and can be completed by people without any knowledge of meditation [38,39]. Studies have demonstrated that mindfulness practice increases an individual’s FMI score [38,40] and that FMI scores are, on average, higher for experienced meditators compared to the FMI scores of non-meditators [41], indicating that the FMI scale is a good tool to validate the consistency of groups of experienced meditators compared to non-meditators [38]. We ensured that, at the group level, FMI scores, and thus trait mindfulness, are higher for experienced meditators than would be expected for non-meditators.

### 1.3. The Choice-Blindness Paradigm

The CB paradigm involves giving participants false feedback on their choices, thus creating a mismatch between their intentions and the outcome of their choices [9,14,15,16,42,43]. CB occurs when participants fail to notice this discrepancy and produce a confabulatory answer when the experimenter asks for the reason why they chose something that they in fact never chose [10,44]. This effect can occur during simple visual choices (e.g., a choice between two pictures) but also during more complex choices, such as the choice between two objects only discriminated by touch [45], or the recognition of a verbal declaration (see [46], in which participants believed they had said something they, in fact, never said).

Two interpretations of the CB phenomenon are of interest here. CB could be the result of insufficient introspection [10,11,12] and of the under-estimation of the influence of environmental and situational factors (environmental factors include the participant’s physical surroundings. Situational factors would instead consist of the general context of the situation they are currently in, such as the fact that they are participating in an online experiment) [13,14,15,16,17]. Yet, an attitude of openness allows meditators to obtain better introspective access [5,6] as well as an improved capacity to focus on environmental details [7,8].

Moreover, positive emotions increase the perception of false feedback in a visual task of CB [47], and the practice of meditation increases well-being and positive emotions [21,48,49]. Therefore, we suggest that mindfulness meditators should be better able to notice the mismatch and thus more easily avoid CB compared to non-meditators.

In this study, we replicated a computer-mediated version of the original paradigm of CB [9,50,51]. As the previously mentioned articles indicate, CB remains observable in a computer-mediated paradigm, though it is less prevalent than in a face-to-face context. For example, the CB effect also remains present when the experiment takes place in a virtual reality environment [52].

As we described above, greater access to state mindfulness could potentially counter CB by remedying insufficient introspection and underestimation of the influences of environmental and situational factors, as the lack of these two aptitudes is the main cause of CB described in the literature.

The main hypothesis is that mindfulness meditators should be less sensitive to the CB effect compared to non-meditators. Thus, we suppose experienced mindfulness meditators will be able to detect more manipulations in this CB context compared to non-meditators. To investigate this, we conducted a comparative and correlational study including two groups: a group of experienced mindfulness meditators (experimental group) and a group of non-meditators (control group). We counted the number of times participants in each group reported detecting a manipulation.

We thus expect to observe a higher rate of manipulation detection in the group of mindfulness meditators compared to the control group.

## 2. Materials and Methods

### 2.1. Participants

A total of 66 participants aged between 25 and 55 participated in the study (14 men, 51 women and 1 who did not identify as either, $M_{Age}=37.7$). Participants were split in two groups following their experience with mindfulness meditation (cf. subsection “Definition of the groups”). Overall, 31 participants ($M_{Age}=39$) were experienced meditators, and 35 ($M_{Age}=36.5$) were non-meditators. All participants had obtained their Baccalauréat (end of high school diploma) or a higher diploma. They were all native French speakers and reported in the questionnaire not having any knowledge of CB. Both groups were given an identical CB task. Participants all gave their informed consent, could stop the experiment at any moment and were debriefed at the end of the experiment. The experimental protocol was approved by the Ethics Committee of the P-A-R-I-S Association (http://paris-reasoning.eu, accessed on 2 November 2022. The ethics committee declaration is available at https://doi.org/10.17605/OSF.IO/37GPY, accessed on 2 November 2022).

### 2.2. Definition of the Groups

The inclusion criteria for the group of experienced meditators were to be a mindfulness meditator practicing multiple times per week for at least 1 year (attention focused on the present moment, meditation focused on breathing, on the body, on thoughts and on the environment), and to have practiced within an institution (association or club). The different types of meditation practices included were mindfulness exercises supervised by an instructor (mindfulness, Vipassana, yoga, Dzogchen). Participants in the group of non-meditators were required to have no experience of mindfulness-related practices, including meditation, yoga, tai chi, qigong or martial arts, but were potentially interested in them. The group formation methodology is similar to those used in several studies [31,53,54,55].

After assigning the participants to one of the two groups (experienced meditators or non-meditators), we checked that the experienced meditators had a higher level of trait mindfulness than non-meditators. To do so, participants answered the validated French version [39] of the Freiburg Mindfulness Inventory (FMI-14 items) measuring trait mindfulness [38] (this allowed us to verify that the group of experienced meditators (N=31) had, on average, a higher FMI score (M=38.7, SD=3.93), and thus stronger trait mindfulness, than that of non-meditators (N=35, M=35.6, SD=5.47) similarly to what can be found in the literature.The two groups were found to be significantly different ($t_{64}=-2.594$, p=0.012), with a medium effect size (with d=0.64). Therefore, meditators had, on average, stronger trait mindfulness than the non-meditators.).

### 2.3. Materials and Procedure

The experiment was a computer-mediated replication of CB paradigms [9,50,51]. Participants used their own computer from their home and had an unlimited amount of time to make their choices, similarly to one condition of the original experiment (in [9], for one condition, participants had 2 s to make their choice, for another, they had 5 s, and for the last condition, they had an unlimited amount of time).

The survey for the experiment was created with the online software Qualtrics. We used greyscale pictures of human faces extracted from the 10k US Adult Faces Database (Copyright 2020 Wilma Bailbridge, https://www.wilmabainbridge.com/facememorability2.html, accessed on 2 November 2022) [56]. The size and greyscale levels of all pictures were normalized. To minimize emotional bias and to respect the original protocol [9], we only selected pictures of young women with neutral faces and formed pairs based on similarities in appearance. Each pair was composed of people of the same ethnicity, age (the average age gap was 4 years), hairstyle, and hair color.

Participants were asked to follow the experiment from home in a quiet place for half an hour (duration of the experiment), during which they should not be disturbed. In order to check if they had been disturbed, they were encouraged to report it and to describe the nature of the disturbance in a free comment space at the end of the questionnaire. No participants reported being disturbed during the experiment.

**Figure 1:**
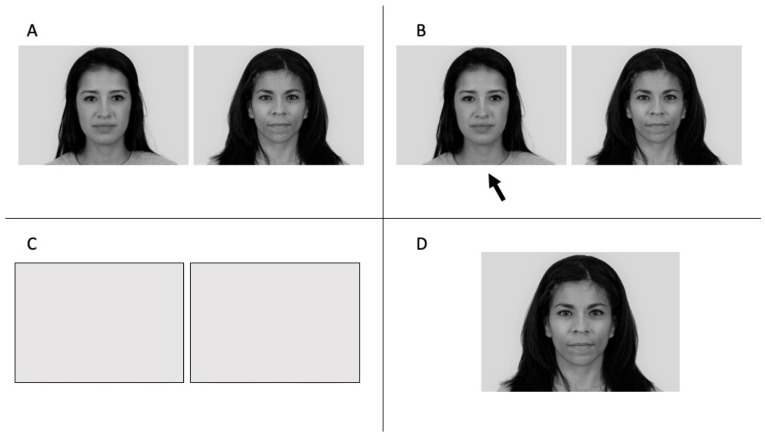
For the M condition (manipulated trials), (**A**) the participant is shown two pictures and is asked to choose the one they find more attractive. (**B**) The participant selects the picture they prefer. (**C**) The two pictures are then hidden. (**D**) The picture supposedly chosen by the participant is displayed, but it is a deception, as the picture shown is in fact the opposite of their choice. Pictures used in our task come from the 10 k US Adult Faces Database. Reprinted with permission from Wilma Bainbridge. Copyright © 2020 Wilma Bainbridge [56].

A total of 15 pairs of grayscale pictures of women’s faces were shown to each participant in random order. Overall, 9 out of the 15 pairs were distractors, and 6 were used to evaluate the participant’s ability to notice that the outcome of their choice was manipulated. Participants had an unlimited amount of time to make their choices. A total of 3 out of these 6 pairs were manipulated (M), and the remaining 3 were not (NM). Once the choice was made, the pictures were hidden. For these 6 pairs (M and NM), the participants were asked to explain their choices. Then, the protocol differed for M and NM. For NM, the chosen picture was then displayed again and disappeared once more prior to this face being rated for attractiveness on a 10-point scale. The other face was not rated. For the M pairs, the picture that did not correspond to their choice was displayed and then rated (Figure 1).

**Figure 2:**
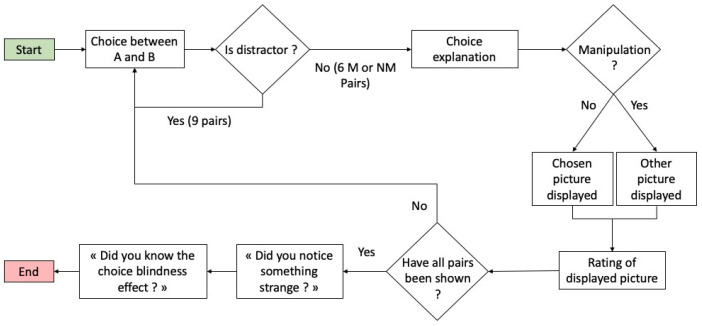
Illustration of the experimental protocol.

Participants were then given the opportunity to freely express themselves. To avoid making the participants suspicious regarding the intent of this open field, the question remained as neutral as possible (“Do you have any remarks or comments to add?” (“Avez-vous des remarques ou commentaires à ajouter”)). These instructions were repeated for all 6 pairs of faces used during the evaluation. Once all 15 pairs had been presented, participants were asked if they had noticed anything strange during the experiment with an open-ended question. Finally, they were asked whether they knew about CB (Figure 2).

### 2.4. Statistical Analyses

We first analyzed the socio-demographic data by performing descriptive statistics to visualize the distribution of the data of the demographic variables (gender, age, level of education and occupation), as well as the means of the FMI score, according to the groups (experienced meditators, non-meditators).

We then performed 2 classical ANOVAs and 1 Bayesian ANOVA to investigate the influence of these variables on manipulation detection (demographic variables and FMI score). Non-significant results would indicate the independence of these variables with respect to the participant’s ability to detect manipulations.

The second analysis concerns the manipulation detection score by group (experienced meditators and non-meditators) expressed in percentages and detection means. In total, the experienced meditator and non-meditator groups were exposed to 93 and 105 manipulations, respectively. We calculated how many manipulations were detected in each group and transformed them into percentages to make the scores comparable.

The third analysis considers the number of manipulations detected per subject (which could be 0, 1, 2, or 3). Two main variables were used in this experiment. The independent variable was the presence or absence of meditation practice. The dependent variable was an ordinal variable with four modalities: the number of detected manipulations.

To find out whether meditators detected more manipulations than non-meditators, we used a multinomial logistic regression for the analysis of the collected data. Two multinomial models were adjusted. First, we adjusted the model in which the linear predictor contained the independent variable (M1, corresponding to a model in which the practice of meditation has a significant influence on the number of manipulations detected), and then we adjusted the null model, which did not contain any predictor (M0, corresponding to a model for the null hypothesis, in which the practice of meditation has no significant influence on the number of manipulations detected).

We then calculated the chances of detecting manipulations as a function of group membership using the odds ratio method.

## 3. Results

### 3.1. Socio-Demographic Analyses

Descriptive analyses were performed on the participants’ socio-demographic data. Table 1 shows the distribution of participants in the non-meditator and meditator groups according to gender, age, level of education, occupation, and average FMI score.

The socio-demographic variables, such as age, sex, education level, and profession, did not have any effect on the detection of manipulations (an ANOVA was run with these variables in the predictive model. Results indicated that sex did not have any effect on the number of detections ($F_{2,44}=1.628,p=0.21$); neither did age ($F_{2,44}=2.227,p=0.11$), nor did education level ($F_{6,44}=0.210,p=0.97$), and nor did profession ($F_{9,44}=0.452,p=0.90$)).

We found no direct influence of FMI scores on the detection of manipulations (with ANOVA) with very strong evidence in favor of the null hypothesis (H0: no effect of FMI scores on the detection of manipulations) ($F_{1,64}=0.484,p=0.489,log\left(BF_{10}\right)=-1.173$).

### 3.2. Percentage of Manipulation Detection

The experienced meditators detected 63.4% of the manipulated trials, which represents 59 manipulations out of 93 ($M_{detection}=1.90$). The non-meditators detected 38.0% of the manipulated trials, which represents 40 manipulations out of 105 ($M_{detection}=1.14$).

### 3.3. Multinomial Models and Odd Ratio

The characteristics of the two multinomial models are shown in Table 2. M1 corresponds to a model in which the experience of meditation has a significant influence on the number of manipulations detected, and M0 corresponds to a model for the null hypothesis, in which the experience of meditation has no significant influence on the number of manipulations detected.

The AIC was higher for M0 than for M1 at 172.2 and 168.9, respectively ($\chi^{2}\left(3\right)=9.26,p=0.026$). Thus, M1 could fit the data better than M0. We can conclude that being an experienced meditator influences the detection of manipulations.

**Figure 3:**
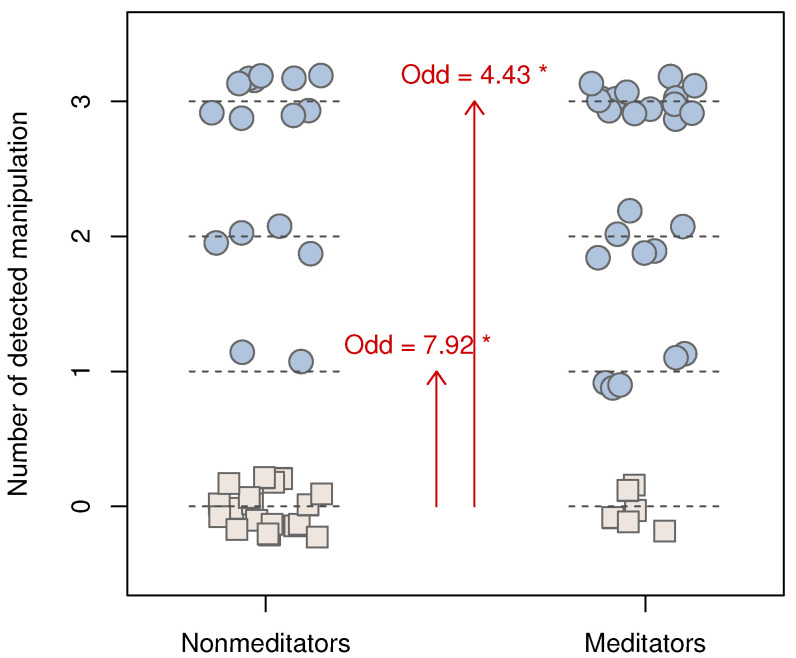
Number of Manipulations Detected by Each Participant Depending on the Condition (non-meditators vs. meditators). Each participant is represented by a symbol aligned with the number of manipulations detected, with some noise added for readability. Square symbols represent participants who did not report any manipulation of their choices (modality used as a reference to estimate the odds ratio of the multinomial models). Red arrows represent significant odds ratios at * p<0.05. R code to reproduce the figure: https://doi.org/10.17605/OSF.IO/37GPY, accessed on 2 November 2022.

The parameters of interest of the model M1 (Table 3) show that the likelihood of detecting a single manipulation (compared to zero) was 7.92 times greater in experienced meditators than in non-meditators (Z=2.16,p=0.031). Detecting three manipulations (compared to zero) was 4.43 times more likely in experienced meditators compared to non-meditators (Z=2.38,p=0.017). We did not find a significant odds ratio regarding the detection of 2 manipulations, but it is close to the significance threshold α of 0.05 (Z=1.95,p=0.051) (Figure 3).

The experienced meditators’ likelihoods (odds ratio) of detecting 1 or more, 2 or more, or all 3 manipulations were 4.95 ($CI_{95}=\left[1.63,15.1\right]$, p=0.005, FET ($CI_{95}$ designates the confidence interval at 95%. The *p*-value is obtained with Fisher’s exact test (FET).)), 4.52 ($CI_{95}=\left[1.44,14.2\right]$, p=0.009, FET), and 4.43 ($CI_{95}=\left[1.30,15.1\right]$, p=0.02, FET) times that of non-meditators. In terms of relative risk, meditators detected 1 or more manipulations 1.76 times more often than the non-meditators (80% and 46%, respectively), 2 or more manipulations 1.61 times more often (65% and 40%, respectively), or all 3 manipulations 1.58 times more often (45% and 29%, respectively).

## 4. Discussion

### 4.1. Interpretations

Results suggest that cultivated mindfulness could influence the detection of manipulations but not dispositional (or trait) mindfulness. CB was reduced for experienced meditators in comparison to what we could observe for non-meditators, as they were more likely to detect manipulations. Furthermore, the socio-demographic variables such as sex, age, profession, and study level cannot explain the better result of the mindful group on the detection of manipulations as there was no statistical difference between the two groups for any of them, leaving only the experience of mindfulness meditation as a predictor of the observed effect on CB.

We believe that the ability of experienced meditators to better detect manipulation can be explained by the habit of meditation because it improves two processes responsible for CB: introspective access [5,6] and a focus on environmental details [7,8], which seems to be associated with a better “scanning” of the environment.

The development of better introspective access can be of great help in countering CB. Indeed, a lack of introspection, which is one of the primary explanations for CB, can be caused either by self-ignorance of our choices or ignorance of the mismatched outcomes [10,11,12]. In the first case, people do not have complete introspective access and fail in the self-assignment of their own choices [10,51]. In the second case, people know their choices and have access to them but are not aware of the fact that their choices have changed. They are also blind to the causes of the changes [10,12,42]. The hypothesis that meditation could improve self-knowledge follows the results from many studies (see [37] for a review). Morris suggests that the many higher mental processes often considered structurally unconscious are actually “pre-conscious” events that would emerge to consciousness only if the person pays attention to them [57]. The results of our study suggest that the repeated practice of mindfulness activities over the course of a lifetime may have helped participants focus their attention on these pre-conscious processes. Thus, the increased performance of meditators could be attributed to the improvement of introspective access due to attention to pre-conscious processes via mindfulness.

Increased well-being in mindfulness meditators [21,48,49] might also explain our results since positive emotions have been shown to reduce CB, likely by enhancing attention [47].

Moreover, mindfulness not only improves one’s ability to focus on environmental details [7,8], but it can also improve mental resilience, increasing people’s ability to scan the context of the experiment on the fringes of what is known [7]. Mindfulness meditators scrutinize themselves with stronger consideration of both success and failure than non-meditators, thereby leading to more effective scanning of the environment for contradictory elements [7]. This could explain why experienced meditators more easily manage to notice the mismatch between their intentions and the outcomes.

Another primary explanation for CB is the under-estimation of the influence of environmental and situational factors in decision-making [13], likely linked to information processing [14,15,17,51]. Thus, thanks to the use of an analytical attitude, mindfulness meditators would be able to consider the importance of the influence of environmental factors during decision-making. This attitude could explain why experienced meditators were more likely to detect manipulations.

It is also possible that experienced meditators had higher detection scores than non-meditators because they acted with more autonomy [27]. Having a high implicit trust in the experimenter who designed the study might indeed inhibit the detection of manipulation [18].

Mindfulness has already been linked to the decreased prevalence of certain cognitive biases [3] that could be involved in CB: the attribution bias, the anchoring bias, and the recognition heuristic. An attribution bias could emerge during the mis-assignment of the manipulated choice (we assign the choice we did not make ourselves). An anchoring bias could be caused by the manipulation (displaying the wrong picture would change future choices). A recognition heuristic could happen when making the choice (this heuristic results from past experiences causing an automated action of choice-making without the reasoning being consciously accessible).

It would thus seem that mindfulness meditators could more easily de-automatize their mental operations [23,58], switching from an autonomous mode to a more analytical mode (respectively, processes of type 1 and type 2, as they are described by Kahneman [59] thanks to an improved ability to opt out of heuristics). The mental dispositions responsible for this de-automatization would be awareness (discontinuing automatic inference processing), attention (enhancing cognitive control), focus on the present moment (facilitating meta-cognitive insight), and acceptance (preventing suppression or thought distortion) [23]. Thanks to these mechanisms, mindfulness meditators should be able to opt out of automatic processes of thinking (type 1 processes) to adopt a more analytical mode of thinking (type 2 processes). These elements could be responsible for the decrease in CB in experienced meditators.

Given that this study is only correlational, it could be argued that an alternative interpretation of our results would be that experienced meditators could have already had a greater ability for introspection even before practicing mindfulness. This predisposition could then have been enhanced and maintained by regular mindfulness practice.

### 4.2. Limitations

This study focuses on a population of self-reported meditators. All participants had to certify that they regularly practiced mindfulness meditation in the context of a club or association to consolidate the meditator group. The higher FMI score of meditators compared to that of non-meditators allows the validation of the groups. However, it would be interesting to reproduce this experiment in a longitudinal study to more strictly control the practice of meditation in order to further strengthen the conclusions drawn from these data.

It is also important to point out that FMI scores are not directly correlated with the better detection of manipulations. This may seem counter-intuitive, as both groups had different FMI scores and different detection rates, but this does not hold at an individual level. This observation highlights the weakness regarding the reliability of the FMI survey in regard to the assessment of the mindfulness trait of individuals.

Moreover, the FMI score seems to be difficult to compare between expert and naive participants. Indeed, this comparison is controversial for two reasons. First is the occurrence of the Dunning–Kruger effect. This effect appears when a lack of expertise in a particular field causes errors in self-assessment [60,61]. We can suppose that some non-meditators overestimated their capacity for mindfulness, while experienced meditators did better in the self-assessment.

The second reason is that the FMI scale is not systematically understood in the same way by meditators and non-meditators [62]. These two reasons could explain the observed effect of previous meditation experience on the rates of detection of the manipulations, while no significant effect was observed on those rates based on an individual’s FMI scores.

Another potential limitation of our study is the disagreement between the model selection criteria (AIC and BIC), which could indicate ambiguous models. Each criterion has a different purpose. AIC aims at selecting the best model which predicts the data, while BIC aims at selecting the true model which generated the data. As a consequence, AIC has the tendency of favoring overly complex models, while BIC has the opposite tendency of favoring overly simple models when the true model is not in the list of tested models. Much debate exists concerning the criterion that should be used, and for what purpose (see [63] for a comparison of AIC and BIC). In this paper, we decided to focus on AIC, as we do not claim that a model containing only the practice of mindfulness as a predictor of the detection of manipulation can really be the true model that generated the data. Moreover, BIC converges toward selecting the true model when the quantity of samples goes toward infinity. With a limited number of samples here, we considered AIC to be the better criterion.

## 5. Conclusions

This study showed that CB was reduced in experienced meditators compared to non-meditators. These results suggest that the experienced meditators possess characteristics not measured by the FMI, which could explain the results obtained. While this experiment does not allow the investigation of which specific characteristics are involved, we hypothesize that they are related to the regular training of attentional control during meditation practice.

To go further, conducting a longitudinal study on meditators would be interesting in order to confirm these results. Indeed, such a study would allow us to see when this difference in CB sensitivity starts manifesting itself: before the first meditations, which would indicate pre-existing characteristics influencing CB sensitivity, early following the first meditations, which would indicate that the mere awareness of these processes would be enough, or only after a certain time with regular meditation, which would indicate an actual training to de-automatize certain processes.

The question of the de-automatization of decision-making through mindfulness and the investigation of the effects state mindfulness and trait mindfulness each have on cognition should also be further investigated in order to consolidate these results.

## Figures and Tables

**Table 1:** Frequencies for socio-demographic variables.

Variables	Non-Meditators	Meditators
Male	7	7
Female	28	23
Other	0	1
25–35 years	20	13
36–45 years	8	9
46–55 years	7	9
BAC	5	1
BTS-DUT	2	2
DUG	0	2
LICENCE	13	9
MAITRISE	4	6
MASTER	9	9
DOCTORAT	2	2
Craftsmen, shopkeepers, company managers	0	1
Executives and higher intellectual professions	4	5
Employee	1	2
Student	12	4
Worker	1	1
Intermediate occupations	14	15
Retired	1	1
Unemployed	1	1
Missing	1	1
FMI score (Mean)	35.6	38.7

**Table 2:** Main Characteristics of the models M1 and M0.

Model Name	Log-Likelihood	Degrees of Freedom	Residual Deviance	AIC	BIC
M1	−78.4	192	156.9	168.9	182.1
M0	−83.1	195	166.2	172.2	178.8

M1 includes the experience of meditation as a predictor, while M0 does not. AIC = Akaike information criteria. BIC = Bayesian information criteria.

**Table 3:** Parameters of Interest of the model M1.

Comparison	Parameter (Odds Ratio)	Std Error	z-Value	*p*-Value
0–1	2.07 (7.92)	0.96	2.16	0.031 *
0–2	1.56 (4.75)	0.80	1.95	0.051
0–3	1.49 (4.43)	0.63	2.38	0.017 *

Each parameter corresponds to the comparison taking as reference the modality 0 (no detection of any manipulation). They were then transformed into an odds ratio. Here, 0–1 indicates the parameter corresponding to going from 0 to 1 detection, and similarly for the two other parameters (0–2 and 0–3). * *p* < 0.05.

## Data Availability

The data and statistical analyses that support the findings of this study are openly available in Choice-Blindness Decreases for Mindfulness Meditators—Data Repository at https://doi.org/10.17605/OSF.IO/37GPY, accessed on 2 November 2022.

## Abbreviations

The following abbreviations are used in this manuscript:

AIC	Akaike Information Criterion
BIC	Bayesian Information Criterion
CB	Choice-Blindness
FMI	Freiburg Mindfulness Inventory
M	Manipulated
NM	Non-Manipulated
